# Intestinal Spirochetosis: To Treat or Not to Treat

**DOI:** 10.7759/cureus.53248

**Published:** 2024-01-30

**Authors:** Kimberly Ho, Joseph Xu, Seymour Katz, Suparna A Sarkar, Ateeqa Mujeeb Ullah

**Affiliations:** 1 Department of Pathology, New York University (NYU) Langone Health, Mineola, USA; 2 Department of Internal Medicine, University of California Davis, Sacramento, USA; 3 Department of Internal Medicine, New York University (NYU) Langone Health, Mineola, USA; 4 Department of Pathology and Laboratory Medicine, New York University (NYU) Langone Health, Mineola, USA

**Keywords:** metronidazole, helicobacter pylori, ulcerative colitis, inflammatory bowel diseases, management, intestinal spirochetosis

## Abstract

Spirochete colonization of the gastrointestinal tract is a poorly understood phenomenon presenting with varying signs and symptoms. Due to the lack of a unified approach and its varying presentations, the management decision for intestinal spirochetosis (IS) has always been challenging. While metronidazole is the commonly preferred antimicrobial treatment, it remains unclear if therapeutic intervention is indicated for everyone, especially asymptomatic patients. We present three patients, diagnosed with IS. They presented with varying demographics, clinical presentations, and past medical histories and underwent different clinical managements. Our decisions for treatment not only included presenting symptoms but also factors like history of pre-existing gastrointestinal diseases, age, and immune status.

## Introduction

Spirochetes, tiny spiral-shaped bacteria sometimes found on the surface of epithelial cells, represents a well-debated conundrum in the gastroenterology and pathology world. Colonization of spirochetes in the lower gastrointestinal tract is a rare condition and can lead to a myriad of presentations, ranging from asymptomatic individuals to symptomatic diarrhea and abdominal pain [1,2]. The mechanism of diarrhea caused by spirochetes is believed to be related to the loss of microvilli, the absorptive surface of the colon, caused by bacterial colonization [3,4]. Due to limited literature availability on its pathogenicity and diverse response to antimicrobial treatment in this subset of patients, guidelines on managing this condition remain open-ended [5-8]. We report three patients, all diagnosed with IS, following colonoscopy and biopsy, all with different presentations, and past medical histories, resulting in different clinical managements.

## Case presentation

Case 1

**Figure 1:**
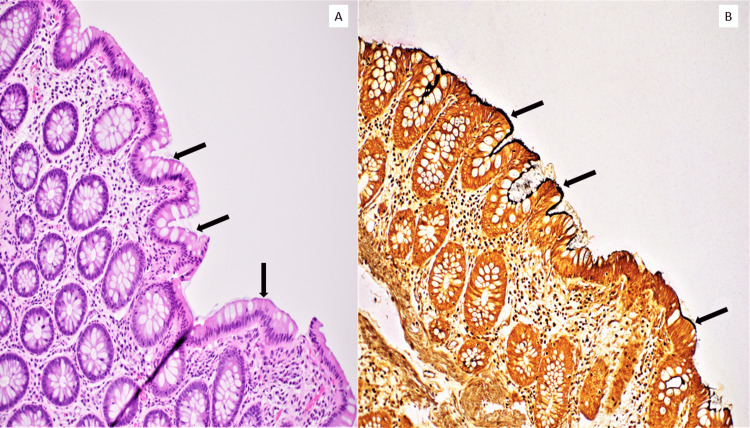
Intestinal spirochetosis as visualized on a colonic biopsy specimen A. Hematoxylin and eosin stain shows a purple, fuzzy "false-brush border" on luminal epithelial cells (arrows) in a colon biopsy specimen. B. Warthin-starry silver stain highlights the bacterial organisms as a black, fuzzy line covering the luminal epithelial cells (arrows).

A 20-year-old male with a history of reflux esophagitis experienced six months of intermittent supraumbilical pain with anorexia and loose mucoid bowel movements. Family history included a parent with irritable bowel syndrome (IBS). His medications included bismuth or famotidine for reflux. Physical examination was significant only for audible borborygmi, but no tenderness or organ enlargement. Esophagogastroduodenoscopy (EGD) was unremarkable. Colonoscopy revealed a small aphthous ulcer in the terminal ileum. Random colonic biopsies were taken that on histopathological examination showed a hyperchromatic, fuzzy, colonic epithelial luminal brush border. A positive Warthin-Starry (WS) silver stain confirmed the diagnosis of IS (Figure 1).

The patient was not immunosuppressed and subsequent laboratory workup for all other infectious pathogens including HIV and inflammatory bowel disease (IBD) was negative. No specific treatment was prescribed initially; however, persistent symptoms prompted treatment with metronidazole. Follow-up colonoscopy with biopsies did not show spirochete colonization.

Case 2

**Figure 2:**
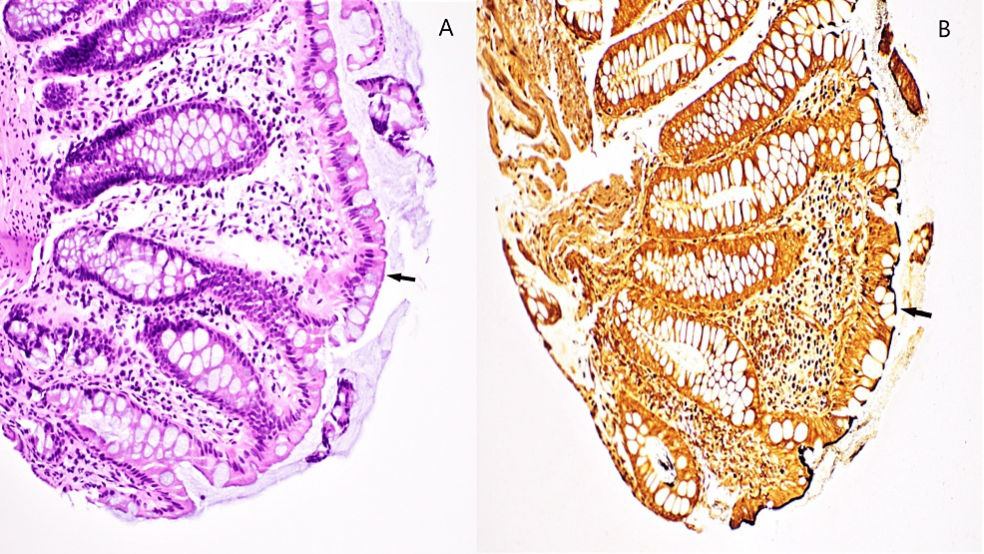
Intestinal spirochetosis in a rectal biopsy A. Hematoxylin and eosin stain with a false-brush border on luminal epithelial cells (arrow). B. Warthin-starry silver stain highlighting the bacterial organisms (arrow).

A 66-year-old male presented to the hospital for an annual screening colonoscopy. He had a past medical history significant for long-standing ulcerative colitis (UC). The patient was not immunosuppressed, and his disease was well controlled on baseline mesalamine maintenance therapy. No active symptoms were reported, and the physical examination was within normal limits. The colonoscopy performed was significant for erythematous mucosa and diverticulosis in the sigmoid colon, two 4 mm polyps in the rectum, and non-bleeding internal hemorrhoids. Multiple biopsies were taken, and the characteristic false brush border was visualized on histology from the descending colon and rectal biopsies, confirmed to be IS by WS silver stain (Figure 2).

No active or chronic colitis was seen microscopically. In the absence of active symptoms, no further investigations for any other pathogens were undertaken, and no antimicrobial treatment was offered. On annual follow-up, the patient reported no new symptoms and health parameters were within normal limits.

Case 3

**Figure 3:**
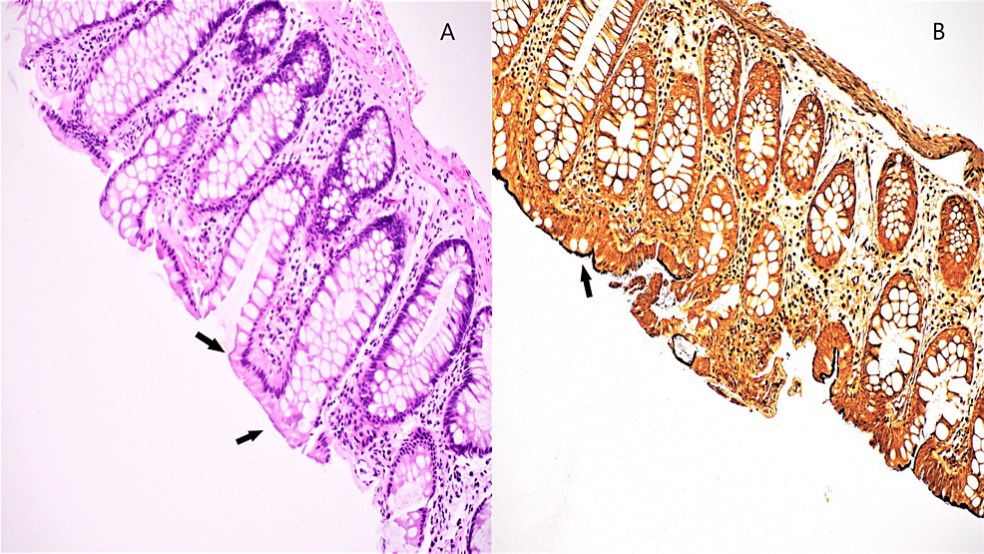
Intestinal spirochetosis seen in a patient with ulcerative colitis A. Hematoxylin and eosin stain with faint, fuzzy appearance on the surface of luminal epithelial cells (arrows). B. Warthin-starry silver stain highlighting diffuse involvement by spirochetes (arrow).

An 82-year-old female with a history of left-sided UC and acute rectal bleeding presented with generalized abdominal discomfort, chills without fevers, and two episodes of nausea and vomiting. A rectal exam was significant for blood mixed with stool and no abdominal tenderness. EGD with gastric biopsies was remarkable for a* Helicobacter pylori* (*H. pylori*) infection. Colonoscopy revealed inflammation characterized by altered vascularity, erosions, erythema, friability, and granularity in a continuous and circumferential pattern from the anus to the rectum, graded as Mayo score 2. Multiple diverticula were found in the sigmoid, descending, and transverse colon. On histopathology, there was moderately active chronic proctitis consistent with idiopathic IBD. Ascending and sigmoid colon biopsies were negative for active colitis, however, significant for the presence of IS, confirmed by WS silver stain (Figure 3).

The active IBD was treated with rectal and oral mesalamine and steroid enema and no other immunosuppressive therapy was offered to the patient. The patient was also treated with quadruple therapy for their *H. pylori* infection that included metronidazole, a treatment strategy also useful for IS. Follow-up was significant for the resolution of symptoms. Oral mesalamine maintenance therapy for IBD was continued.

## Discussion

IS is defined as the presence of filamentous non-treponemal spirochetes, *Brachyspira aalborgi* or *Brachyspira pilosicoli* on the surface epithelium of the gastrointestinal tract, commonly the large intestine. The prevalence of IS varies from 1.1 to 5% in developed countries, and the infection is thought to spread by the fecal-oral route [1]. The prevalence increases up to 54% in homosexual men and HIV-positive individuals, suggesting sexual transmission in some cases [7]. Clinical presentation varies significantly, and individuals may be asymptomatic or present with non-specific symptoms including abdominal pain, diarrhea, and rectal bleeding [1-3]. In our case series, IS was detected incidentally in two of three (Cases 2 and 3) patients, both of which had past medical history positive for IBD. None of the patients were immunosuppressed or had other infectious pathogens including HIV. The sexual orientations of the patients are not known. 

The gold standard method for diagnosis of IS is histopathological assessment of the biopsy specimen with the organism carpeting the surface of the luminal epithelium. Differential diagnosis includes entero-adherent gram-negative *Escherichia coli, *a prominent glycocalyx, and artifacts from biopsies done on a poorly prepped colon. Spirochetes can be highlighted by silver stains like Steiner or WS stain [4] or immunohistochemical stains for *Treponema pallidum* which cross-reacts with *Brachyspira* species. Other newer methods of diagnosing IS include using polymerase chain reaction targeting genes specific to the *Brachyspira* genus, immunomagnetic separation, and fluorescent in situ hybridization with oligonucleotide probes against 16S rRNA gene of *Brachyspira* spirochetes [6-10]. However, none of these methods are currently used clinically [11].

The proposed mechanism of spirochete-mediated intestinal injury includes the erosion of the microvilli of the gut surface epithelium [3]. Pathogenic factors include bacterial sialidases, which cleave sialic acid residues in epithelial oligosaccharides, glycoproteins, and glycolipids to derive energy and evade the immune system. Spirochetes also have collagenase to facilitate bacterial colonization of the extracellular matrix components and a multidrug efflux pump system, conferring antibiotic resistance and survival in the gastrointestinal tract [4].

Metronidazole is commonly the preferred antimicrobial treatment of intestinal spirochetosis as reported in the literature; however, there are no formal treatment guidelines for IS [1]. Older studies have reported that metronidazole may be the drug of choice in eradicating IS [6,10]. However, based on newer research, a “wait-and-watch” approach with close follow-up is gaining popularity as an equally beneficial mode of management, especially for asymptomatic and IBS patients [7]. Alternatively, one study reported that metronidazole treatment in IBS patients paradoxically caused spirochetes to invade crypts and goblet cells, thereby evading antibiotic treatment and increasing disease severity [8].

In cases with concurrent IBD, antimicrobial treatment with metronidazole is preferred over active monitoring [12] as research suggests that patients with concurrent gastrointestinal disease (for example IBD, *H. pylori*-associated gastritis, polyps, etc.) may have an increased risk of spirochete infection due to elevated inflammatory mediators. For example, IBD increases baseline inflammation in the intestinal mucosa, predisposing to a greater immune response to spirochete colonization. These patients are also more likely to present with symptoms including abdominal pain, loss of appetite, nausea, and vomiting. IBD has also been associated with extra colonic lesions, increasing the severity of infection [9,13,14].

Further research is needed to understand the pathogenicity of IS and the risk factors that contribute to spirochete colonization. This understanding would aid in the development of more effective treatment guidelines and regimens. In the interim, healthcare providers could utilize algorithmic approaches to manage patients with IS [15], while considering factors such as age, history of pre-existing gastrointestinal diseases, and immune status. This would enable providers to make informed decisions about the necessity of metronidazole treatment further improving patient care.

## Conclusions

IS poses a management conundrum because of limited understanding of the disease and diverse clinical presentation. There are no formal treatment guidelines available in the literature, both for symptomatic and asymptomatic patients. Where few older studies have reported metronidazole as the drug of choice in eradicating IS, newer research suggests that a “wait-and-watch” approach with close follow-up may also be equally beneficial, especially for asymptomatic patients. In conclusion, further research is warranted to understand the pathogenicity and predisposing risk factors of spirochete colonization, thus, improving and establishing treatment guidelines for IS.
